# Biology and Ultrastructural Characterization of Grapevine Badnavirus 1 and Grapevine Virus G

**DOI:** 10.3390/v14122695

**Published:** 2022-11-30

**Authors:** Martin Jagunić, Angelo De Stradis, Darko Preiner, Pierfederico La Notte, Maher Al Rwahnih, Rodrigo P. P. Almeida, Darko Vončina

**Affiliations:** 1 Department of Plant Pathology, University of Zagreb Faculty of Agriculture, 10000 Zagreb, Croatia; 2 National Research Council of Italy, Institute for Sustainable Plant Protection, Via Amendola 122/D, 70126 Bari, Italy; 3 Department of Viticulture and Enology, University of Zagreb Faculty of Agriculture, 10000 Zagreb, Croatia; 4 Centre of Excellence for Biodiversity and Molecular Plant Breeding, 10000 Zagreb, Croatia; 5 Foundation Plant Services, University of California-Davis, Davis, CA 95616, USA; 6 Department of Environmental Science, Policy and Management, Rausser College of Natural Resources, University of California, 130 Mulford Hall, Berkeley, CA 94720, USA

**Keywords:** grapevine badnavirus 1, grapevine virus G, real-time PCR, transmission modes, host range, cytopathology

## Abstract

The biological characteristics of grapevine viruses, such as their transmission and host range, are important for the adoption of successful prophylaxis strategies. The aim of this study was to investigate the traits of two newly described grapevine viruses widely distributed in Croatia, grapevine badnavirus 1 (GBV-1) and grapevine virus G (GVG). The vine mealybug (*Planoccocus ficus*) proved to be a vector of GBV-1 and GVG capable of vine-to-vine transmission with overall experimental transmission rates of 61% and 14.6%, respectively. Transmission was also demonstrated by grafting, with an overall transmission rate of 53.8% for GBV-1 and 100% for GVG, as well as by green grafting using the T-budding technique. Symptoms of GBV-1 and GVG were not observed on the woody cylinders of the indicators LN 33, Kober 5BB, 110 Richter and cvs. Chardonnay and Cabernet Sauvignon. Seed transmission and mechanical transmission were not confirmed. Electron microscopy revealed accumulation of GBV-1 particles and viroplasms in the cytoplasm, but no alternations of the cell structure. Infection with GVG revealed the proliferation of tonoplast-associated vesicles inside phloem cells and cell wall thickening.

## 1. Introduction

Viruses are considered one of the greatest challenges in agriculture, especially in vegetatively propagated crops. Grapevine (*Vitis* spp.) is one of the oldest cultivated plants, with worldwide production wherever climatic conditions are favorable. Because of its multiple uses (fresh consumption, raisins, wines, distillates, natural juices, jams, etc.), the grapevine industry represents an important economic sector in many countries. According to the International Organization of Vine and Wine (OIV), over 7.3 million hectares were planted with vines worldwide in 2019 [1], of which 21,220 hectares were in Croatia [2].

Apart from the fact that over 86 different viruses can infect grapevines [3], the intensive exchange of planting material has created the conditions for their global spread. In addition, some grapevine viruses can be transmitted locally by various vectors (nematodes, aphids, scale insects and mites), further contributing to the complexity of their control. However, virus spread can be controlled by adopting preventive measures, of which the production and use of virus-free planting material from clean stocks followed by virus diagnosis and vector control are the most important [4]. Such measures are carried out for some economically important grapevine viruses through sanitary selection programs and certification schemes [5]. Such measures in the European Union (EU) are defined by Regulation of European Commission EU 2019/2072 according to protocols established by the European Plant Protection Organization (EPPO) [6], but regulated viruses may differ depending on the country [4]. Nevertheless, the spread of other non-regulated viruses is still a major concern, especially those that have been discovered more recently, primarily using high-throughput sequencing (HTS).

In this context, grapevine virus G (GVG) was first reported by HTS in New Zealand in 2017 [7], then in Croatia in 2018 [8] and next in the USA in 2019 [9]. As for other members of the genus *Vitivirus*, its positive-strand RNA genome contains five open reading frames (ORFs) encoding a polyprotein, a 154-amino-acid-long protein, a movement protein, the coat protein (CP) and a viral nucleic acid binding protein [7]. A large-scale survey conducted in Croatia in 2020–2022 on 4357 grapevines detected the presence of GVG in 77 commercial vineyards in the coastal viticultural region and in three grapevine collections, resulting in an overall infection rate of 10.5%. Interestingly, infections were confirmed only in cultivars considered autochthonous of Croatia and showed a high CP sequence similarity among isolates originating from the same site/vineyard [10]. Although some members of the genus *Vitivirus* are economically important viruses, grapevine virus A (GVA) and grapevine virus B (GVB) have been well characterized in terms of cytopathology, modes of transmission and epidemiology [11], such data are still unknown for GVG. Similarly, the grapevine-infecting members of the genus *Badnavirus* have all been discovered with HTS [8,12,13]. Grapevine vein clearing virus (GVCV) is the first badnavirus infecting grapevine and the first DNA virus of grapevine discovered in the USA [12]. GVCV, associated with vein clearing and vine decline disease, can lead to severe losses and vineyard clearing [14]. In addition, it is graft- and vector-transmissible by the aphid *Aphis illinoisensis* and is known as one of the most important emerging viruses whose distribution is, so far, limited to the USA [15]. On the other hand, the second discovered grapevine-infecting badnavirus was identified in Greece on cv. Roditis and named grapevine Roditis leaf discoloration-associated virus (GRLDaV) [13]; GRLDaV had some detrimental effects on some grapevine cultivars and was shown to be transmitted by the vine mealybug (*Planococcus ficus* Sign.) [16,17]. It was included in the EPPO alert list, mainly to prevent the risk of spread in Europe. Nevertheless, after the first report, the virus was reported in Italy, Croatia, Turkey and South Africa [8,18,19,20]. The third badnavirus infecting grapevine was reported in Croatia in 2018 and named grapevine badnavirus 1 (GBV-1) [8]. The genome organization of this virus consists of three ORFs encoding a hypothetical 33.4 kDa protein, a polypeptide with similarity to reverse transcriptase; ribonuclease H, cauliflower mosaic virus peptidase (A3), a zinc-binding motif; and a hypothetical 35.6 kDa protein. GVB-1 has so far been detected only in grapevines from Croatia with an infection rate of 13.4% [21], predominantly in autochthonous grapevine cultivars grown in the coastal wine-growing region.

After an initial investigation to develop robust detection methods, perform partial molecular characterization, and uncover data on the prevalence of GBV-1 and GVG in Croatian viticultural regions [10,21], the aim of this study was to provide information on the transmission routes, the host range, symptoms in commonly used indicator plants, and cytopathology in grapevines. The data provided will be a prerequisite for the development and implementation of management strategies to prevent further spread.

## 2. Materials and Methods

### 2.1. Virus Transmission by the Vine Mealybug (Planoccocus ficus Sign.)

Virus transmission experiments for GBV-1 and GVG were performed under greenhouse conditions using first- and second-stage instars of the vine mealybug (*P. ficus*). The mealybug colony was established on butternut squash (*Cucurbita moschata* Duch.) and tested by the PCR method for species confirmation [22]. As a source of GBV-1 and GVG inocula, grapevine accession cv. Plavac mali infected with GBV-1 and cv. Vlaška infected with GVG and grapevine leafroll-associated virus 3 (GLRaV3) were used. The sanitary status was previously determined by real-time PCR-based protocols for GVA, GVB [23], grapevine fleck virus (GFkV) [24] and grapevine leafroll-associated viruses 1 (GLRaV1) and -3 (GLRaV3) [25,26], while for grapevine fanleaf virus (GFLV) and arabis mosaic virus (ArMV), primers and probes designed at Foundation Plant Services (UC Davis—personal communication) were used.

The recipient plants were grapevine (*V. vinifera*) seedlings of cvs. Grk × Panonia and Žlahtina, developed from seeds taken from uninfected grapevines. For their production, berries from source plants, previously tested for GBV-1 and GVG, were harvested at physiological maturity (end of September). Before stratification, the seeds were surface-sterilized with a 5% solution of Izosan G (Pliva, Zagreb, Croatia). For the stratification, the seeds were placed in moist sand and kept in a refrigerator at 5 °C for 72 days. Afterward, the seeds were placed on moist filter paper in Petri dishes in a growth chamber for germination at 25 °C (16 h of light). After germination, the seeds were disinfected for 15 min with a 5% Izosan G solution, then treated for 4 h with a 5% solution of a Plant preservative mixture (PPM; Plant Cell Technology, Washington, DC, USA) and sown in polystyrene containers with a substrate under greenhouse conditions (12 h of daylight at 25 °C; 12 h dark at 19 °C). Finally, the seedlings were transplanted into pots with the same substrate and used for transmission experiments at the stage of 3–5 fully developed leaves.

In addition, the herbaceous test plants *Chenopodium murale* L. and *Nicotiana benthamiana* D., as well as annual weeds commonly found in Croatian vineyards, redroot pigweed (*Amaranthus retroflexus* L.), annual ragweed (*Ambrosia artemisifolia* L.), white goosefoot (*Chenopodium album* L.) and gallant soldier (*Galinsoga parviflora* Cav.), were used in vector transmission experiments. Plants were grown from the seeds of mother plants previously tested for GBV-1 and GVG. Before sowing, in *A. retroflexus*, *A. artemisifolia* and *C. album,* the dormancy was interrupted with a 2% KNO_3_ solution for 24 h.

The vector transmission experiments were conducted over two consecutive years with a 48 h acquisition access period (AAP) and a 48 h inoculation access period (IAP) using 10 instars per plant. Afterward, the IAP recipient plants were sprayed with an imidachloprid-based insecticide and maintained under greenhouse conditions. Three months after inoculation, they were tested for GBV-1 and GVG using the real-time PCR protocols described below.

### 2.2. Mechanical Inoculation

Petioles collected from GBV-1- and GVG-infected source vines (described above) were ground and homogenized in a mortar and then diluted in a ratio of 1:10 (weight:volume) using three different buffers: phosphate (0.01 M, pH 7), nicotine (2.5% in distilled water) [27] and phosphate–nicotine–cysteine buffer (0.01 M K_2_HPO_4_, 0.01 M cysteine HCl and 3% nicotine solution in distilled water) [28]. The homogenates were inoculated at the stage of 3–5 fully developed leaves, according to the standard protocol [27], onto grapevine seedlings (*C. murale*, *N. benthamiana*, *A. retroflexus*, *A. artemisifolia*, *C. album* and *G. parviflora*) and velvetleaf (*Abuthilon theophrasti* Medik.). Grapevine seedlings, herbaceous test plants and weeds were grown from seeds according to the above-described protocols. The number of inoculated plants depended on the number of well-developed plants after cultivation. The experiment was carried out over two consecutive years in the greenhouse, and recipient plants were tested by real-time PCR three months after inoculation.

### 2.3. Seed Transmission

GBV-1-positive grapevine cvs. Stara brajda, Galac crni, Gustopupica, Pavicić, Mekuja, Oskorušica and Krstičevica and GVG-positive grapevine cv. Žlahtina were used as seed sources for seedling production. The procedure for obtaining seedlings from GBV-1- and GVG-infected mother plants was the same as already described for seedlings from non-infected vines. All seedlings that reached the stage of 3–5 fully developed leaves were tested for the presence of GVB-1 and GVG by real-time PCR using the protocols described below.

### 2.4. Graft Transmission

During dormancy, grapevine canes were collected from commercial vineyards from vines infected with GBV-1 and GVG. After 48 h of soaking in fungicide (0.15% Teldor SC 500—Bayer AG, Leverkusen, Germany; 0.2% Proplant—Agriphar S.A, Ougrée, Belgium), buds were top-grafted on four different virus-free indicators: *Vitis rupestris*, *Vitis riparia*, Kober 5BB (*Vitis berlandieri* × *Vitis riparia*) and LN 33 (Couderc 1613 × *Vitis berlandieri*). Stratification was conducted in wet sawdust for one month; afterward, the grafted cuttings were planted in 3 L pots filled with a mixture of Steckmedium and TS-2 medium (Klasmann-Deilmann, Geeste, Germany). Fourteen months after planting, petioles were collected from indicators and tested for GBV-1 and GVG using real-time PCR.

### 2.5. Green Grafting Using T-Budding Technique

Grapevine seedlings infected with GBV-1 and GVG+GLRaV3 through vector transmission experiments were used as the source of buds for green grafting. Green buds were collected in May and grafted onto rooted cuttings of five indicators, Kober 5BB, LN 33, 110 Richter (*V. berlandieri × V. rupestris*), *V. rupestris*, *V. riparia*, and cvs. Chardonnay and Cabernet Sauvignon, in 3 replicates. The grafted indicators were kept under greenhouse conditions for five months. Afterward, leaf petioles developed on grafted indicators were collected and tested for the presence of GBV-1 and GVG by real-time PCR. Subsequently, symptoms were inspected by visual observations of the leaves and, after bark removal, of woody cylinders.

### 2.6. Electron Microscopy

Grapevine seedlings single-infected with GBV-1 and co-infected with GVG and GLRaV3 were selected for ultrastructural characterization. Selected leaves were cut into two parts, with one half used for virus confirmation through real-time PCR and the other half used for electron microscopy. For thin sectioning, tissue pieces from mesophyll tissues of GBV-1-infected grapevine leaves and from the main veins of expanded leaves of the GVG- and GLRaV3-infected vines were processed according to standard procedures, i.e., fixation in 4% glutaraldehyde in 0.05 M phosphate buffer for 2 h, post-fixation in 1% osmium tetroxide for 2 h, staining overnight in 0.5% aqueous uranyl acetate, dehydration in ethanol and embedding in Spurr’s medium [29]. Thin sections were stained with lead citrate and observed under a Philips Morgagni 282D electron microscope (Philips, Amsterdam, The Netherlands).

### 2.7. Detection by Real-Time PCR Assays

For the detection of the investigated viruses in all plants, nucleic acids were isolated according to a previously described protocol [30] and used for virus detection using protocols based on real-time PCR for GBV-1 [21], GVG [10] and GLRaV3 [26]. Real-time PCR reactions were performed on a 7500 Real-Time PCR System (Applied Biosystems, Thermo Fischer Scientific, Waltham, MA, USA) with the quantitation cycle (Cq) threshold set at 30.

## 3. Results

### 3.1. Vector Transmission

In 2020, GBV-1 and GVG were successfully transmitted by first- and second-stage instars of the vine mealybug (*P. ficus*) from infected rooted cuttings to 9 of 20 (45%) and 4 of 20 (20%) grapevine seedlings, respectively. Since no weeds or herbaceous test plants were infected with either virus, only grapevine seedlings were used in the experiments conducted in 2021 to more precisely determine the transmission rates. In 2021, GBV-1 was detected in 16 out of 21 (76.2%) seedlings, representing an overall infection rate of 61% over a period of two years. The overall infection rate for GVG was 14.6%, as the virus was successfully transmitted to 4 of 20 (20%) grapevine seedlings in 2020 and 2 of 21 (9.5%) in 2021. Since the GVG-source plant used was co-infected with GLRaV3, positive seedlings were additionally tested for GLRaV3 by real-time PCR. As expected, in all GVG-positive seedlings, the presence of GLRaV3 was also confirmed. A detailed overview of the vector transmission results is reported in Table 1.

### 3.2. Mechanical Inoculation

After mechanical inoculation experiments using three different extraction buffers (phosphate, nicotine and phosphate–nicotine–cysteine buffer) in two consecutive years, none of the 26 herbaceous test plants, 43 weed plants and 7 grapevine seedlings used in 2020, as well as 27 herbaceous test plants and 46 weed plants used in 2021, were found to be positive for GBV-1 or GVG (Supplementary Table S1).

### 3.3. Seed Transmission

Seeds from GBV-1- and GVG-infected vines yielded 420 and 100 seedlings, respectively, that reached the stage of 3–5 fully developed leaves. Although collected from seven different grapevine varieties in the case of GBV-1 and from five different accessions of cv. Žlahtina in the case of GVG, none of the tested seedlings gave a positive result in real-time PCR, so seed transmissibility could not be confirmed for either virus (Supplementary Table S2).

### 3.4. Grafting

For both viruses, transmission to the indicators Kober 5BB, *V. rupestris*, LN33 and *V. riparia* was confirmed in both single and mixed infections. The infection rates of GBV-1 varied from 44.4% to 100% in single infections depending on the indicator used, but the infection rates of GVG were 100% for all indicators. Slightly higher rates were observed for co-infections of GBV-1 with GVG in Kober 5BB and LN 33 than for single infections. Overall, the indicators tested showed that the viruses examined were graft-transmissible at a rate of 53.8% for GBV-1 and 100% for GVG. Thus, all American species and hybrids used proved to be hosts for both viruses (Table 2).

### 3.5. Green Grafting

Using infected green buds, GBV-1 was successfully transferred to cvs. Chardonnay and Cabernet Sauvignon by T-budding green grafting, but not to the other indicators used. In contrast, in addition to its transmission to Chardonnay and Cabernet Sauvignon, GVG was also successfully transferred to the indicators LN 33, Kober 5BB and 110 Richter.

**Figure 1:**
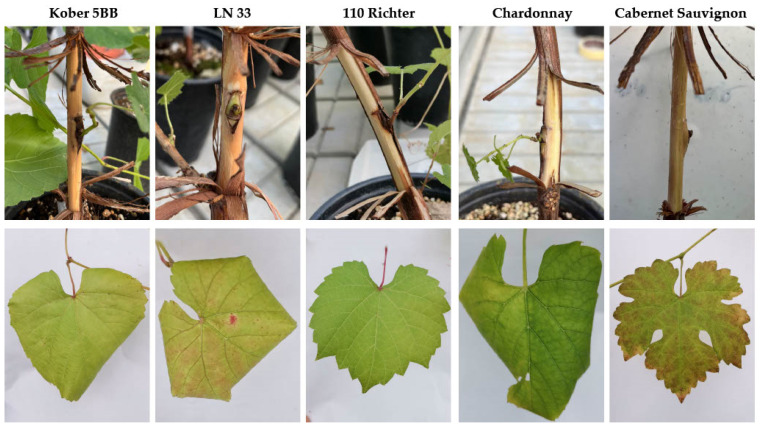
Different symptoms of infection with GVG and GLRaV3 observed on leaves of indicators Kober 5BB, LN33, Chardonnay and Cabernet Sauvignon five months after green grafting, with the exception of asymptomatic 110 Richter. Related woody cylinders show no symptoms of infection with GVG and GLRaV3.

Five months after grafting, in the case of GBV-1, no changes or symptoms associated with virus infections were observed on leaves or woody cylinders after bark removal. Although no changes were observed on the woody cylinders of plants infected with GVG+GLRaV3, leafroll-like symptoms were evident on the leaves of all of the indicators tested, with the exception of 110 Richter. Symptoms such as downward leaf rolling and yellowing were observed on Kober 5BB and Chardonnay, reddening and leaf rolling were observed on LN 33, while only reddening was observed on Cabernet Sauvignon (Figure 1).

### 3.6. Electron Microscopy

#### 3.6.1. Ultrastructural Characterization of GBV-1-Infected Grapevine

**Figure 2:**
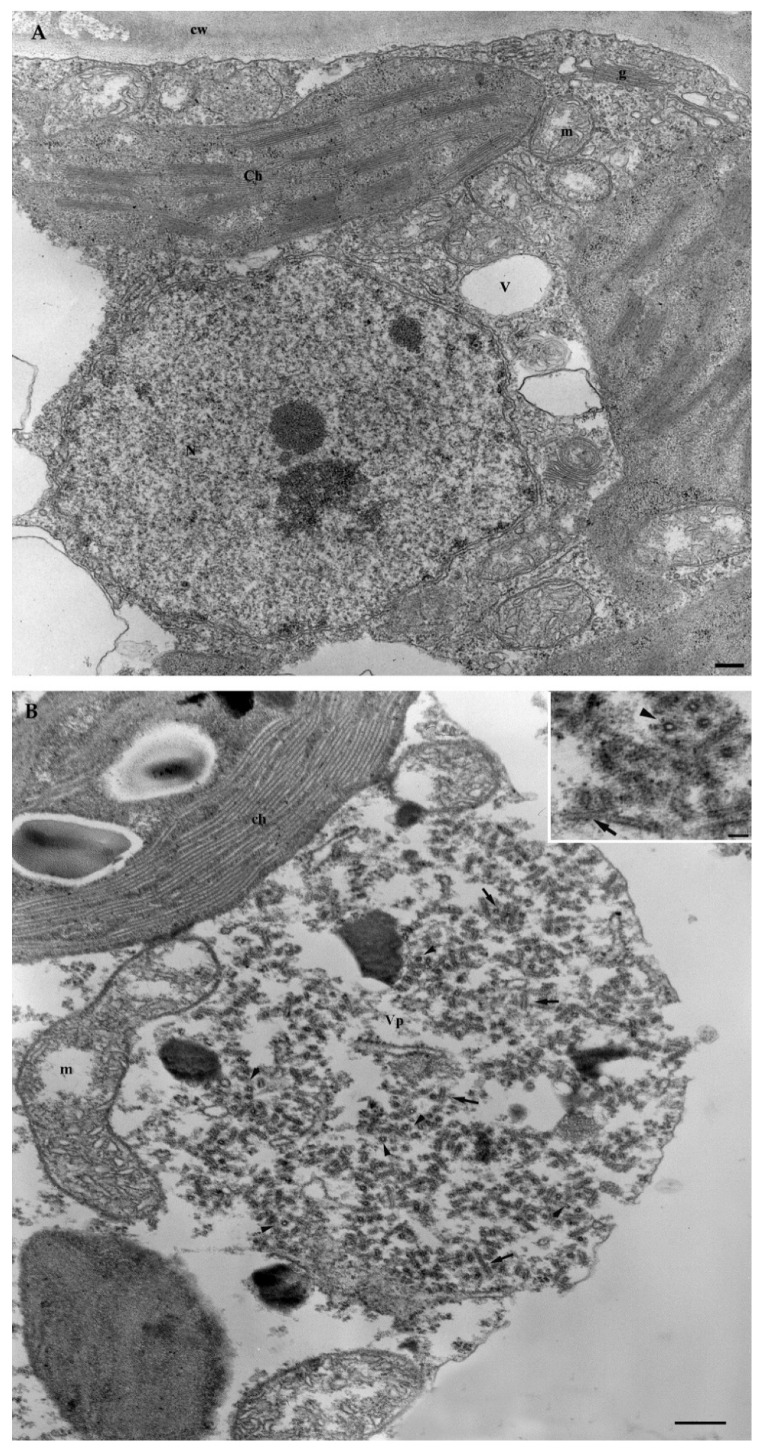
(**A**) Mesophyll cell showing the normal appearance of the cytoplasm and organelles. N = nucleus; Ch = chloroplast; V = vacuole; m = mitochondria; g = Golgi; cw = cell wall. Bar = 250 nm. (**B**) Ultrastructure of GBV-1-infected leaves, particularly that of the viroplasm in a mesophyll cell, where virus particles assemble and accumulate. Inset shows GBV-1 particles sectioned in the transverse (arrowhead) and longitudinal (black arrows) planes. Vp = viroplasm; ch = chloroplast; m = mitochondria. Bar = 250 nm; inset = 50 nm.

The structural architecture of infected cells was well preserved as compared with the healthy control (Figure 2A). Virus particles were plentiful, appearing as round or bacilliform structures, depending on whether they were cross- or longitudinally sectioned, and occurred in aggregates of various sizes (viroplasms), either scattered in the cytoplasm or in the proximity of major organelles (Figure 2B).

#### 3.6.2. Ultrastructural Characterization of GVG+GLRaV3-Infected Grapevine

**Figure 3:**
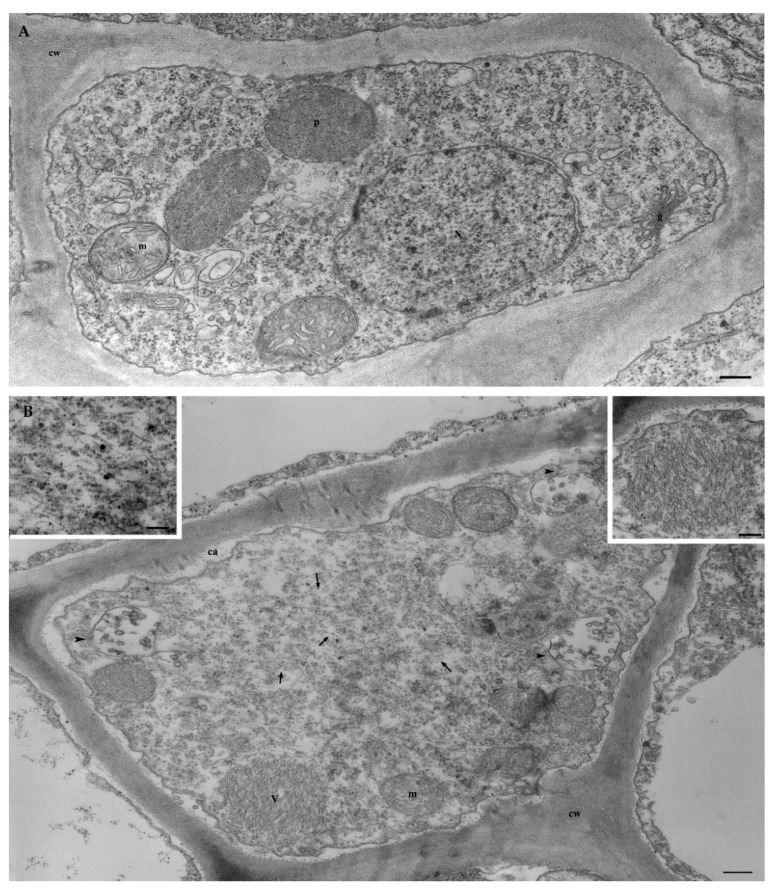
(**A**) Virus-free phloem cell showing the normal appearance of the cytoplasm and organelles. N = nucleus; m = mitochondria; p = peroxisome; g = Golgi; cw = cell wall. Bar = 250 nm. (**B**) Cell in a vascular bundle of vine co-infected by GVG and GLRaV3 shows scattered fine particles (arrows and enlarged left inset) and tonoplast or cell membrane-associated vesicles (arrow-heads) for the vitivirus GVG, and an aggregate of virus particles (V) for the ampelovirus GLRaV3 (enlarged right inset). Massive appositions to the cell wall (cw) of callose-like material (ca) have been observed. m = mitochondria. Bar = 250 nm; inset = 100 nm.

The cytopathology of vines co-infected with GVG and GLRaV3 was characterized by the presence of scattered viral particles of both viruses in the cytoplasm of phloem cells compared with the virus-free control (Figure 3A), which showed a normal organelle architecture. GVG appeared in the form of thin filaments causing the proliferation of vesicles derived from the tonoplast and cell membrane (Figure 3B). 

## 4. Discussion

Studies on the impact, biology and epidemiology of recently discovered grapevine viruses, mainly using HTS, are important in determining the real threat to viticulture and, consequently, developing and implementing control strategies to stop or slow down their spread. Such studies have already confirmed some vitiviruses and badnaviruses as economically important pathogens for viticulture. Recently, studies on GBV-1 and GVG in Croatia have shown their wide distribution in autochthonous cultivars from the coastal wine-growing region, with overall infection rates of 13.4% and 10.5%, respectively [10,21]. Considering the fact that both viruses were found mainly in autochthonous germplasm and are not regulated in the certification scheme, there is a risk of their further spread. The active spread of grapevine viruses over short distances is most effectively mediated by various vectors, whose monitoring and control are the most important measures to prevent the virus from spreading in newly established vineyards. Since four grapevine vitiviruses (GVA, GVB, GVE and GVH) [31,32] and a grapevine-infecting badnavirus (GRLDaV) have been shown to be transmitted by scale insects [17], the same type of vector was selected to study the transmission of GBV-1 and GVG. In our experiment, the vine mealybug (*P. ficus,* Hemiptera: *Pseudococcidae*), a common pest of grapevines [33], proved to be a vine-to-vine vector of both viruses (GBV-1 61%; GVG 14.7%). Previous studies showing very high infection rates with specific viruses at different sites (up to 96% for GBV-1 and 100% for GVG) and high genetic similarity among the isolates at each site [10,21] suggested possible local transmission. Although the risk of the further vector-mediated spread of the virus in regions where *P. ficus* occurs is obvious, further studies should focus on other potential vector species, such as mealybugs and soft-scale insects, which are not typical just in Mediterranean regions. This would provide additional data on the risk of spread on a global scale.

In addition to grapevine, other plant hosts may also play an important role in the biology and epidemiology of grapevine viruses, as has been noted in various cultivated plants and wild hosts that can potentially serve as inoculum reservoirs [34,35]. In addition to woody hosts, weeds in vineyards may also be infected with grapevine viruses but may also serve as food for vectors, such as dagger nematodes [36]. Grapevine badnaviruses and vitiviruses have not yet been reported in weeds, which was also confirmed by our study, where transmission to weeds commonly found in Croatian vineyards (*C. murale*, *N. benthamiana*, *A. retroflexus*, *A. artemisifolia*, *C. album*, *G. parviflora* and *A. theophrasti*) by either the vine mealybug or mechanical inoculation was not successful. However, weeds in vineyards have already been found to be infected with grapevine fanleaf virus (GFLV; family *Secoviridae*, genus *Nepovirus*), which was confirmed in *Aristolochia clematitis* and *Lagenaria siceraria* in Hungary [37] and in *Cynodon dactylon, Polygonum* spp., *Sorghum halepense, Melilotus* spp. and *Plantago lanceolata* in Iran [38,39,40]. More recently, studies on the host range of grapevine Pinot gris virus (GPGV) have shown that it is present in several weeds commonly found in Italian vineyards: *Ailanthus*, *Asclepias*, *Crataegus*, *Fraxinus*, *Rosa*, *Rubus* and *Sambucus* [41]. These results revealed previously unknown sources of virus inocula and suggest the need to revise the current management strategies for some viral diseases.

In contrast to cultivated plants and weeds, which are commonly used for host range studies, laboratory herbaceous test plants have been used for the characterization and detection of grapevine viruses. Since herbaceous hosts can support virus replication better than grapevine, they are used for in-depth studies [42]. GVA, GVB and GVD have been identified in *Nicotiana* species after mechanical inoculation [28,43,44,45] but can also be transmitted by mealybugs, such as *P. ficus* [46,47], *Pseudococcus longispinus* [46], *P. affinis* [48] and *Parthenolecanium corni* [49]. In addition to vitiviruses, GRLDaV has been shown to be mechanically transmissible to *Chenopodium quinoa, Gomphrena globosa, N. benthamiana*, *N. tabacum*, *N. rustica* and *Physalis floridana* [13,16]. Based on these findings, our attempts to transmit GBV-1 and GVG to the herbaceous test plants *N. benthamiana* and *Ch. murale* by mechanical inoculation and vectors (*P. ficus*) were not successful.

Since grapevine planting material is produced vegetatively, generative propagation does not represent a major risk of virus transmission and spread. However, the seed transmission of grapevine viruses may be of interest for breeding programs. In this study, this mode of transmission was not confirmed for GBV-1 and GVG, as all 420 and 100 seedlings, respectively, developed from the seeds of infected mother plants tested negative. These results are consistent with a previous study on rugose-wood-associated viruses (GVA, GVB and grapevine Rupestris stem pitting-associated virus—GRSPaV), viruses from the leafroll complex (GLRaV1, 2 and 3), ArMV, GFLV and GFkV [50]. However, in that study, the results for GRSPaV and GFLV were inconsistent because seed transmission had already been documented [51,52]. In contrast, more recent studies on GLRaV2 and GPGV confirmed the possible seed transmission from mother plants infected with the aforementioned viruses [53]. In addition, GRSPaV has been shown to be transmitted to seedlings of Cabernet Sauvignon, but not when the seeds of cvs. Muscadelle and Pinot noir were used [54]. All of this suggests that the seed transmission of grapevine viruses is highly dependent on the grapevine cultivar used, as previously suggested [53]. Therefore, the data on the seed transmission of GBV-1 and GVG should be supplemented in the future by studies with other cultivars.

Today, trade in planting stock is the major route for the spread of grapevine viruses over long distances [3,4]. Consequently, graft transmission is an important factor in the epidemiology of grapevine viruses. In this study, the graft transmission ability was demonstrated to be 44.4% and 100% for GBV-1 and GVG, respectively. Unlike other viruses, which are considered significant and regulated by certification schemes, in the case of newly discovered viruses, there is no such regulation, so the possibility of their further spread increases accordingly. Since both viruses are unregulated in Croatia, the national and international exchange of planting material, especially of autochthonous grapevine cultivars, poses a serious risk for their further dissemination.

To gain better insight into the effect of GBV-1 and GVG on grapevine, green grafts were made on woody indicators routinely used in grapevine virus testing. Unfortunately, the transfer of GBV-1 from the pure source to the indicators used was not successful, but it was successfully transferred to cvs. Chardonnay and Cabernet Sauvignon. In both cultivars, no changes in woody cylinders or leaves were evident five months after grafting, raising the possibility of latent infections. A potentially neutral effect of GBV-1 on grapevine may be supported by a recent study, in which the virus was found in several species of figs (*Ficus* spp.) in Russia [55], raising the possibility that grapevine is not the primary host of GBV-1. The possibility of transmission from figs to grapevines is very likely, since most vineyards traditionally contain or are near fig trees.

In contrast to GBV-1, GVG was successfully transferred to grapevine cvs. Chardonnay and Cabernet Sauvignon and to the indicators Kober 5BB, LN 33 and 110 Richter. While no changes were evident in the woody cylinders, symptoms were present in the leaves of all plants except 110 Richter. Unfortunately, the cleanest GVG source was one co-infected with GLRaV3, and the symptoms observed resembled those typical of grapevine leafroll disease (GLD; downward leaf curling, yellowing/reddening) [56]; GLD-like symptoms described in Shiraz disease (SD) reported in South Africa and Australia were associated with GVA, with the absence of GLRaV3 confirmed by HTS [57]; a co-infection relationship and even synergy with ampeloviruses have already been suggested for vitiviruses, as they were frequently found together, with higher vitivirus populations and more pronounced symptoms [58], as well as simultaneous transmission with mealybugs [59]. Such synergy was associated with plant death in SD in South Africa, which was not observed in SD-symptomatic GLRaV3-free vines in Australia [60]. This study supports the concept of co-infection being important, as all GVG-positive vines were also infected with GLRaV3. In view of this, GVG might be involved in the GLD symptoms observed in GVG+GLRaV3-infected indicators/vines or even cause such symptoms in the absence of GLRaV3, which needs to be demonstrated in further studies.

Because herbaceous plants were not infected after transmission experiments, grapevine seedlings were used as the cleanest available sources of GBV-1 and GVG for ultrastructural characterization by electron microscopy. GBV-1-infected seedlings were used as a single-infected material, whereas GVG-infected seedlings were co-infected with GLRaV3, as co-transmission occurred. Although GBV-1 was found as typical baciliform particles that form aggregates in different cultures, like other members of the genus *Badnavirus* [61], no significant cytological alterations were observed in the infected plant cells. In contrast, the typical cytopathology of vitiviruses and ampeloviruses was observed in grapevine cells infected with GVG+GLRaV3. GVG was associated with the formation of vesicles derived from the tonoplast, which was previously confirmed in *Nicotiana* cells infected with GVA, GVB and GVD [28,45,62]. In addition, wall thickening, which has been previously reported in GVA [62,63] and GVD [45], probably as a result of the accumulation of callose-like substances [11], was also observed in this study and may be related to GVG, as this effect has not been reported for GLRaV3. Other effects, such as the proliferation of membranes or changes in organelles reported for GVA and GVD, were not observed in this study. In addition, the typical vesiculation of mitochondria by GLRaV3 was observed, as previously reported [64].

In summary, GBV-1 and GVG are viruses transmitted from vine to vine by an insect vector (*P. ficus*) and by green grafting. Both viruses are graft-transmissible to the indicators *V. riparia, V. rupestris*, Kober 5BB, and LN 33. Since no symptoms were detected on the leaves or woody cylinders of cvs. Chardonnay and Cabernet Sauvignon, and no structural changes were observed in infected cells, the role of GBV-1 as a pathogen remains unclear. On the other hand, since Kober 5BB and LN 33, together with grapevines infected with GVG and GLRaV3, showed leafroll-like symptoms, the role of GVG in the symptomatology remains to be clarified. Ultrastructural analyses of GVG-infected cells showed typical vitivirus cytopathology. No alternative hosts were found in the main weeds found in Croatian vineyards or in the herbaceous species tested as indicators. This study provides valuable data on the biology and epidemiology of GBV-1 and GVG that may be useful in design strategies to limit their further spread.

## Figures and Tables

**Table 1:** Results of vine mealybug (*P. ficus*) transmission of GBV-1 and GVG using different recipient plants.

Virus	Year	Recipient Plants	Species	No. of Infected Plants/No. of Used Plants (%)
GBV-1	2020	Herbaceous test plants	*Chenopodium murale*	0/10
			*Nicotiana benthamiana*	0/20
		Weeds	*Amaranthus retroflexus*	0/10
			*Ambrosia artemisifolia*	0/20
			*Chenopodium album*	0/20
			*Galinsoga parviflora*	0/10
		Grapevine seedlings	*Vitis vinifera*	9/20 (45%)
	2021	Grapevine seedling	*Vitis vinifera*	16/21 (76.2%)
GVG	2020	Herbaceous test plants	*Chenopodium murale*	0/10
			*Nicotiana benthamiana*	0/20
		Weeds	*Amaranthus retroflexus*	0/10
			*Ambrosia artemisifolia*	0/20
			*Chenopodium album*	0/20
			*Galinsoga parviflora*	0/10
		Grapevine seedlings	*Vitis vinifera*	4/20 (20%)
	2021	Grapevine seedlings	*Vitis vinifera*	2/21 (9.5%)

**Table 2:** Graft transmission results for GBV-1 and GVG.

Virus	Indicator	No. of Positive/No. of Grafted Plants (%)	
GBV-1	Kober 5BB	4/9 (44.4%)	
	*Vitis rupestris*	1/1 (100%)	
	LN 33	1/2 (50%)	
	TOTAL	6/12 (50%)	
GVG	Kober 5BB	36/36 (100%)	
	*Vitis rupestris*	3/3 (100%)	
	LN 33	4/4 (100%)	
	*Vitis riparia*	6/6 (100%)	
	TOTAL	49/49 (100%)	
GBV-1+GVG	Kober 5BB	GBV-1	4/7 (57%)
		GVG	7/7 (100%)
	*Vitis rupestris*	GBV-1	1/2 (50%)
		GVG	2/2 (100%)
	LN 33	GBV-1	2/3 (66.7%)
		GVG	3/3 (100%)
	*Vitis riparia*	GBV-1	2/2 (100%)
		GVG	2/2 (100%)
	TOTAL	GBV-1	9/14 (64.3%)
		GVG	14/14 (100%)

## Data Availability

Not applicable.

## Supplementary Materials

The following supporting information can be downloaded at: https://www.mdpi.com/article/10.3390/v14122695/s1. Table S1: Results of the mechanical inoculation tests for GBV-1 and GVG using different herbaceous test plants, weeds, and grapevine seedlings. Table S2: Results of the seed transmission trials for GBV-1 and GVG.
